# Establishment of immortalized Schwann cells derived from rat embryo dorsal root ganglia

**DOI:** 10.3892/ijmm.2012.1016

**Published:** 2012-06-06

**Authors:** HUAJUN JIANG, WEI QU, FENG HAN, DAZHUANG LIU, WEIGUO ZHANG

**Affiliations:** Department of Orthopaedics, First Affiliated Hospital of Dalian Medical University, Dalian 116011, P.R. China

**Keywords:** Schwann cells, human telomerase reverse transcriptase, nerve growth factor, brain-derived neurotrophic factor, transfection

## Abstract

Schwann cells (SCs) play an important role in the development, function and regeneration of peripheral nerves. They can enhance both peripheral and central nerve regeneration by providing a supportive environment for neurite outgrowth through the release of neurotrophic factors. However, use of primary SCs for *in vitro* models is limited because these cells are difficult to prepare and maintain in high yield and purity under common cell culture conditions. Human telomerase reverse transcriptase (hTERT) expression induces immortalization of various cell types without substantial alterations of their phenotypes. Therefore, in this study we transfected SCs with hTERT to establish a reliable cell source and observed the effect of hTERT on SCs. In order to accomplish this, SCs were isolated from rat embryo dorsal root ganglions, transfected with hTERT at early passage (passage 3). SCs passage 4, 8, 12 and 30 after transfection (hTERT-SCs) were used for immunocytochemistry, RT-PCR and western blotting. Results showed that all the early (passage 4) and late (passage 30) passage hTERT-SCs expressed hTERT mRNA and gained full telomerase activity. The transfection did not alter the mRNA expression of senescence-associated genes, such as p53 and p16. The expression of BDNF (brain-derived neurotrophic factor) was significantly decreased as cell passage increased, compared to the untransfected control. On the other hand, the expression of NGF (nerve growth factor) was elevated at early passages (passages 4 and 8) and decreased at late passages (12 and 30). These data indicate that the use of specific immortalization techniques can establish SC lines that retain characteristics of typical primary SCs, and different mechanisms responsible for regulating NGF and BDNF expression. This is the first report regarding the immortalization of SCs derived from rat embryo dorsal root ganglions. These cells are useful in studies investigating the cellular mechanisms and regenerative processes of SCs.

## Introduction

Schwann cells (SCs), the principal supporting cells of the peripheral nervous system, play a key role in peripheral nerve regeneration. Autologous nerve grafting or biodegradable conduits are the gold standard for peripheral nerve repair (1). Use of primary somatic SCs for peripheral nerve repair is limited because these cells are difficult to prepare and maintain in high yield and purity under common cell culture conditions.

Transfection of an exogenous hTERT, encoding the catalytic subunit of human telomerase, can be used to prevent telomere shortening, overcome telomere-controlled senescence, and immortalize primary somatic cells. Most importantly, hTERT alone can immortalize cells without causing cancer-associated changes or altering phenotypic properties. Ectopic hTERT expression induces immortalization of adult canine SCs (2) and primary human fetal SCs (3) without substantial alterations of their phenotypes. Moreover, the nerve regeneration process is also regulated by neurotrophic factors (NTFs) synthesized directly or indirectly by SCs. Therefore, it is essential to evaluate the secretion of NTFs after SCs are exposed to an exogenous hTERT. However, there is limited reports on the relationship between the secretion of NTFs and the transfection of hTERT.

In this study, we describe how the induction of the hTERT gene allowed us to establish immortalized SCs derived from rat embryo dorsal root ganglions. Additionally, we evaluated whether these cell lines have SCs properties, and examined the function of the secretion of NTFs (BDNF and NGF).

## Materials and methods

### Cell culture

A total of 30 healthy Sprague-Dawley female rats were obtained by the Animal Experimental Center of Dalian Medical University (License No. SCXK(Liao)2006-0002). Dorsal root ganglions (DRG) from rat embryos were isolated under a microscope. Non-nervous tissue was gently stripped under a dissecting microscope, and the nerve segments were stored in ice-cold Dulbecco’s modified Eagle’s medium (DMEM) containing 1% penicillin/streptomycin. Briefly, dissected dorsal root ganglions were digested with 0.3% collagenase type I solution (Sigma, USA) and 0.25% trypsin (Sigma). After blocking the enzymatic digestion with DMEM containing 10% fetal bovine serum (Gibco, USA), cells were centrifuged and subsequently mechanically dissociated. At ∼75% confluence after plating, cells were purified with 2 cycles of cytosine arabinoside (10 mM) to eliminate fibroblasts and neurons (4). Finally, the media was changed with melanocyte growth medium (Cascade Biologics, Portland, OR) to which 2 μM forskolin (Sigma), 10 ng/ml FGF-2 (Sigma) and 5 μg/ml bovine pituitary extract (Sigma) were added in order to prevent fibroblast proliferation (5). All cultures were maintained at 37°C in a humidified atmosphere of 5% CO_2_. The growth medium was renewed every 2–3 days. SCs were subcultured to the next passage when reaching 80–90% confluence.

### Transfection of the hTERT gene

When SCs (passage 3) reached 90% confluence, transfection was performed. SCs were transfected with pCI-neo-hTERT plasmid (Yrbio, China) using Lipofectamine™2000 (Invitrogen, USA) according to the manufacturer’s instructions. After selection with 400 μg/ml G418 (Sigma), the resistant clones were picked and expanded under standard conditions with 100 μg/ml G418. The growth medium was a melanocyte growth medium containing 2 μM forskolin (Sigma), 10 ng/ml FGF-2 and 5 μg/ml bovine pituitary extract. SCs were subcultured to the next passage when which on reaching 80–90% confluence.

### Indirect immunofluorescence staining

For immunocytochemistry, hTERT-SCs (passage 30) cultured on coverslips were fixed with 4% paraformaldehyde (PFA) in PBS for 10 min and then permeabilized with 0.05% Triton X-100 for 10 min. Non-specific sites were blocked with 5% goat serum for 1 h. Then, the cells were incubated with primary antibodies for 12 h at 4°C. The following primary antibodies were used rabbit anti-S100β (Boster, Wuhan, China), mouse anti-p75 (Boster), mouse antiglial fibrillary acidic protein (anti-GFAP, Boster) all at 1:100 dilution. Blocking solution without a primary antibody was added for 12 h at 4°C as a control. Antibody binding was detected by using fluorescently labeled secondary antibodies (Vector Laboratories, Burlingame, CA) at 1:200 dilution and cell nuclei were counterstained with 4′,6-diamidino-2-phenylindole dihydrochloride (DAPI,1:1000). The coverslips were then washed three times in PBS (5 min each), and finally, the cells were mounted in antifade solution (6). Labeled cells were examined with fluorescence microscopy. The images were digitally recorded and processed using Image-Pro Plus.

### Detection of telomerase activity

hTERT-SCs of passages 4, 8, 12, and 30 and SCs of passage 3 were extracts corresponding to 10^4^ cells, and the telomerase activity was evaluated in these clones using the TRAP assay according to the manufacturer’s instructions as previously suggested (7). PCR reaction products were separated on 12.5% non-denaturing acrylamide gels. After fixation [0.5 M NaCl, 50% ethanol, and 40 mM sodium acetate (pH 4.2)], the gels were directly exposed to an X-ray film with an intensifying screen.

### Reverse transcriptase polymerase chain reaction (RT-PCR)

hTERT-SCs (5×10^4^/ml) of passages 4, 8, 12, and 30 and SCs (5×10^4^/ml) of passage 3 were trypsinized and seeded onto 6-well plates. When cells reached 90% confluence, RT-PCR was performed. Total RNA was harvested using Trizol reagent (Invitrogen). Total RNA (200 ng) was reverse transcribed for 45 min at 60°C, after which a two-step PCR amplification was performed, according to the manufacturer’s instructions. mRNA from cDNA samples was amplified with specific primer pairs for β-actin, hTERT, NGF-β, BDNF, p16, and p53. The primer sequences and the PCR conditions are depicted in Table I. Expression of hTERT, p53 and p16 in hTERT-SCs were examined at passage 4 and 30; HT-29 (HeLa, ATCC:CCL-218) were used as positive controls. The secretion of NTFs (BDNF and NGF) were examined at hTERT-SCs of passages 4, 8, 12 and 30, and untransfected SCs of passage 3 were used as control cells. β-actin expression was detected as the internal control. Amplification products were separated by 2.0% agarose gel electrophoresis and visualized by ethidium bromide staining. After the gels were scanned, the relative intensity of the bands was determined by using the LabWorks 4.6 gel imaging and analysis software (UVP, Inc.). The experiments were repeated three times.

### Western blotting

To confirm the results from the secretions of NTFs (BDNF and NGF), we detected the secretions of NTFs (BDNF and NGF) at SCs of passages 4, 8, 12 and 30 after transfection respectively using western blot analysis; untransfected SCs of passage 3 were used as control cells. SCs were washed with PBS and lysed with a lysis buffer (1% Triton X-100, 150 mM NaCl, 10 mM Tris, pH 7.4, 1 mM EDTA, 1 mM EGTA, pH 8.0, 0.2 mM Na_3_VO_4_, 0.2 mM PMSF, 0.5% Nonidet P-40) and incubated on ice for 30 min. The cell lysates were then clarified by centrifugation at 9,000 x g for 10 min at 4°C and the supernatant was saved for protein analysis and western blotting. Total protein concentration was determined by using a commercially available kit based on the bicinchoninic acid (BCA) method. Total protein lysates (80 μg) were separated by 10% SDS-PAGE mini-gel. Samples were transferred electrophoretically to nitrocellulose membranes. The membrane was blocked with 5% nonfat dry milk in Tris buffered saline with Tween-20 (TTBS), washed with TTBS and incubated overnight with anti-active NTFs (BDNF and NGF) (1:500) and β-actin antibody (1:2000, Sigma). After washing in TTBS, the bound antibody was detected through the use of avidin-conjugated horseradish peroxidase (1:500, Sigma). Color reaction was obtained using NBT/BCIP. The membranes were scanned and analyzed using the LabWorks 4.6 software. The experiments were repeated three times.

### Statistical analysis

Results are expressed as mean ± SEM (standard error of mean) of three independent experiments. One-way ANOVA followed by the Newman Keuls multiple comparison test was used to compare control and treated groups, with P<0.05 indicating significant differences.

## Results

### Transfection of SCs, expression of hTERT, p53 and p16

**Figure 1.**
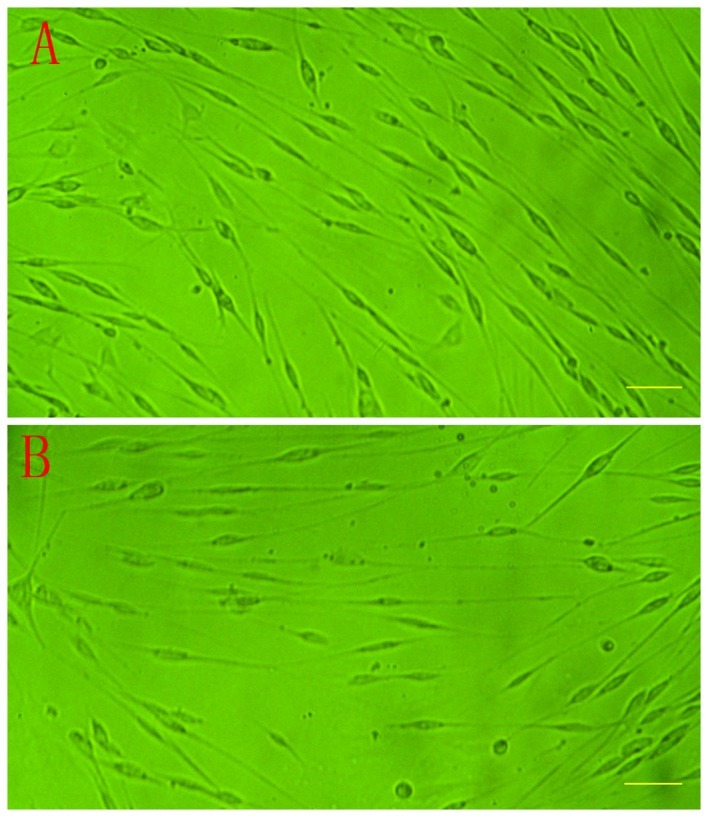
Phase-contrast photomicrographs of cultured cells. (A) Population of SCs after purification. (B) Population of hTERT-SCs after selection with 400 μg/ml G418. Bars, 50 μm.

After the second purification procedure, the primary SCs cultured within 2 weeks reached 90% confluence (Fig. 1A). Cells at early passage (passage 3) were transfected with the CI-neohTERT plasmid construct expressing the catalytic subunit of the hTERT and a selection of positive clones was conducted in G418 (100 μg/ml) containing medium. Over 100 G418-resistant clones were isolated from each experiment. However, the doubling time of hTERT-SCs was not significantly modified after transfection (Fig. 1B).

**Figure 2.**
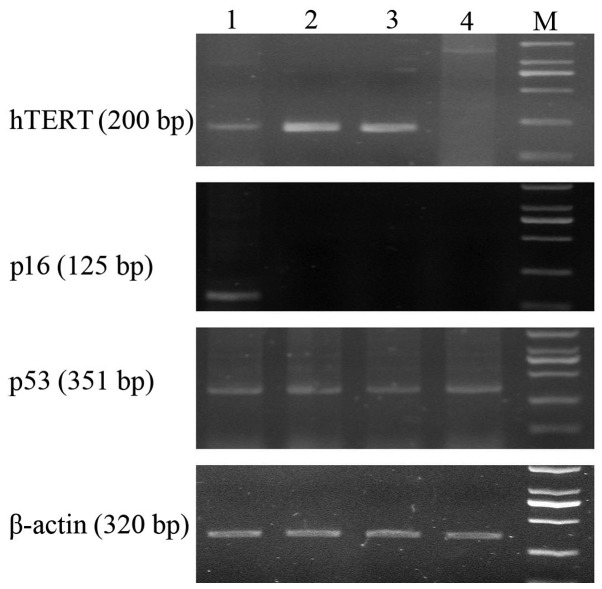
RT-PCR analysis of hTERT expression and p53, p16 expression in SCs. Lane 1, HT-29 cells served as positive controls for hTERT and p53/p16, respectively. Lane 2, hTERT-SCs at early passage (passage 4). Lane 3, hTERT-SCs at late passage (passage 30). Lane 4, SCs before transfection. M, marker. β-actin was used as a cDNA qualitative control. All hTERT-SCs were positive for hTERT independent of the passage level. All hTERT-SCs expressed p53 but lacked p16.

All the early (passage 4) and late (passage 30) passages hTERT-SCs expressed hTERT mRNA. hTERT-SCs expressed p53 but lacked p16 as demonstrated by RT-PCR. In addition, p53 and p16 expression was not altered in early and late passages hTERT-SCs compared to their non-transfected counterparts as demonstrated by RT-PCR (Fig. 2).

### Telomerase activity

**Figure 3.**
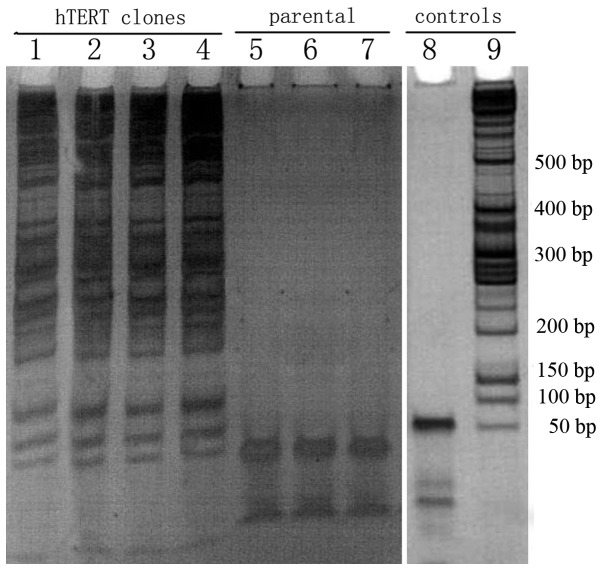
hTERT expression confers telomerase activity to hTERT-SCs. Clones were analyzed for telomerase activity by the TRAP assay. Lanes 1-4, clones isolated from hTERT-SCs of passages 4, 8, 12 and 30, respectively. Lanes 5-7, parental non-transfected cells. Lane 8, negative PCR control. Lane 9, DNA marker.

hTERT-SCs were expanded from each passage (passages 4, 8, 12 and 30) and the telomerase activity was detected in these clones using the TRAP assay. Telomerase activity was detected in all hTERT-SCs passages and these cells displayed full telomerase activity (Fig. 3). This activity was absent in control clones (parental non-transfected cells).

### Immunostaining

**Figure 4.**
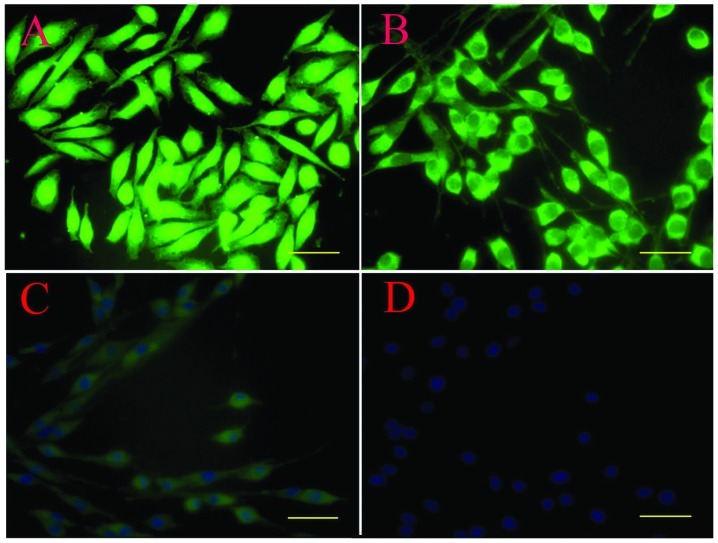
Immunostaining for p75, GFAP, S-100 antibodies. Staining was evident in the cell body and along the processes. hTERT-SCs *in vitro* demonstrated typical bipolar or tripolar morphology and were stained brightly. (A) p75, (B) GFAP, and (C) S-100 staining. (D) Cell nuclei were counterstained by DAPI. Bars, 50 μm.

By immunostaining, hTERT-SCs at passage 30 still showed a positive staining for GFAP, p75 and S100β. The staining was evident in the cell body and along the processes. These cells demonstrated typical bipolar or tripolar morphology *in vitro,* brightly stained for p75 (Fig. 4A), GFAP (Fig. 4B) and S100β (Fig. 4C), and had oval nuclei (Fig. 4D).

### NGF and BDNF mRNA expression detected by RT-PCR

**Figure 5.**
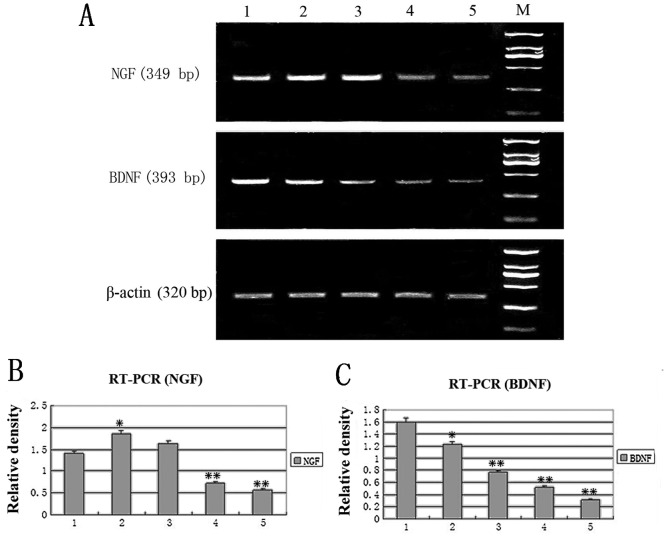
RT-PCR analysis of NGF and BDNF. (A) Lane 1, untransfected control cells. Lanes 2–5, hTERT-SCs of passages 4, 8, 12 and 30, respectively. M, marker. The relative density of (B) NGF and (C) BDNF mRNA vs. β-actin mRNA respectively(^*^P<0.05,^**^P<0.01). Lane 1, untransfected control cells. Lanes 2–5, hTERT-SCs of passages 4, 8, 12 and 30, respectively. M, marker.

We studied the effect of hTERT on the biological activities of SCs by examining NGF and BDNF mRNA through the expression RT-PCR. A semi-quantitative RT-PCR was adopted to analyze the expression of NGF and BDNF mRNA (Fig. 5). The relative density of a 349 bp product of the NGF gene, a 393 bp product of the BDNF gene respectively and a 320 bp product of the internal control β-actin was calculated after separation by 1% agarose gel electrophoresis. Results showed that the expression of the BDNF gene in the transfected SCs was dramatically decreased as cell passage increased, compared to the untransfected control, while the expression of NGF was elevated at early passages (4 and 8) and decreased at late passages (12 and 30). The alteration of gene expression of NGF (Fig. 5B) and BDNF (Fig. 5C) in the transfected SCs was passage-dependent.

### NGF and BDNF protein expression detected by western blotting

**Figure 6.**
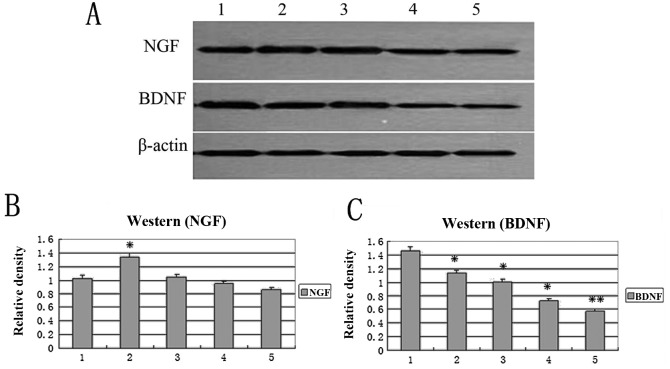
Western blot analysis of NGF and BDNF. Lane 1, untransfected control cells. Lanes 2–5, hTERT-SCs of passage 4, 8, 12 and 30 respectively. The relative density of (B) NGF and (C) BDNF mRNA vs. β-actin mRNA respectively(^*^P<0.05,^**^P<0.01). Lane 1, untransfected control cells. Lanes 2–5, hTERT-SCs of passages 4, 8, 12 and 30 respectively. M, marker.

To detect the protein expression level of BDNF and NGF in the transfected SCs, western blotting was employed (Fig. 6). Results revealed that the expression level of BDNF BDNF gene in the transfected SCs was dramatically decreased as cell passage increased, compared to the untransfected control, while the expression level of NGF was elevated at early passages (4 and 8) and decreased at late passages (12 and 30). In Fig. 6, western blot analysis further confirmed that the alteration of gene expression of NGF (Fig. 6B) and BDNF (Fig. 6C) in the transfected SCs was passage-dependent.

## Discussion

Schwann cells (SCs) are neural crest derivatives that ensheathe and myelinate axons of peripheral nerves. They wrap individually around the shaft of peripheral axons, forming myelin sheaths along segments of the axon. SCs play an important role in the development, function and regeneration of peripheral nerves. They can enhance both peripheral and central nerve regeneration by providing a supportive environment for neurite outgrowth through the release of neurotrophic factors (8–10) and cellular matrix protein (11,12). In addition, SCs promote axonal regeneration and remyelination following transplantation into the lesioned nervous system (13,14). Compared to the use of a conduit alone, artificial nerve grafts containing SCs is a promising method for peripheral nerve repair (15). However, for the application of SCs in tissue engineering or for clinical use, the production of a large number of viable SCs is necessary. Expansion and maintenance of SCs in culture have been difficult, due to the low division rate and potential overgrowth of fibroblasts over time (16–18).

We have demonstrated how the transfer of an exogenous hTERT, encoding the catalytic subunit of human telomerase, can be used to prevent telomere shortening, overcome telomere-controlled senescence, and immortalize primary somatic cells. The introduction of the hTERT gene into primary somatic cells results in telomere length elongation and in the extension of the *in vitro* replicative lifespan of primary somatic cells, then increase cellular proliferation. The major advantage of using hTERT exclusively to immortalize primary somatic cells is that the enzyme telomerase can immortalize without causing cancer-associated changes or altering phenotypic properties (19–21). Adult canine Schwann cells SCs (2) and primary human fetal SCs (3) have been immortalized by hTERT or/and SV40 large T antigen.

In the present study, we used human telomerase reverse transcriptase (hTERT) transfection of postnatal SD rats Schwann cells derived from DRG to establish a stable source for cell transplantation. The morphological phenotypes of transfected SCs were not altered. All the early (passage 4) and late (passage 30) passage hTERT-SCs expressed hTERT mRNA and gained full telomerase activity. This may indicate that SCs were successfully transfected. By immunostaining, the hTERT-SCs at passage 30 still showed a positive staining for S100β, p75, and GFAP without altering phenotypic properties. This indicates that the transfected SCs retained the properties of a primary SCs yet they were easy to grow and propagate. In addition, p53 and p16 expression was not altered in early and late passage transfected SCs compared to their non-transfected counterparts. This may indicate that rat SCs undergo senescence through a telomere-dependent pathway similar to human cells as previously described (22,23).

Many studies introduced hTERT at a time when telomeres had reached a critical length and hTERT expression enhanced cellular proliferation (24–27). One important feature of SCs as a promising cell type for transplantation is their ability to produce a variety of trophic factors that are growth-promotive (28). It was demonstrated that SCs expressed NGF (29), and BDNF (30), and NT-3 (31). Among these factors, members of the neurotrophin family play particular roles. However, little is known about the effects of hTERT treatment on the secretion of SCs.

Based on our results hTERT increased the mRNA level of NGF at early passage, whereas an adverse effect occurred on the mRNA levels and protein secretion of BDNF in SCs at early passages (4 and 8). These results indicated that hTERT is able to enhance the biological activities of SCs through different effects on NGF and BDNF, and hTERT induced different mechanisms responsible for regulating NGF and BDNF expression. This study is similar to the effect of Hypoxia/Reoxygenation on SCs (32), which demonstrated that the time-course and spatial pattern of both expression are distinctly different. After transfection, early cell passages that increased NGF may be correlated with protecting SCs, and stimulating SCs retrodifferentiation and proliferation. However, the function of BDNF, such as inducing SCs and expression of myeline proteins, is not similar to NGF (33). SCs exposed to hTERT, upregulated NGF to meet an urgent requirement of protecting SCs, and downregulated BDNF, a non-urgent requirement. A decrease in BDNF and subsequent decrease in NGF may be correlated with either hTERT-SCs secretion decrease or the decrease in the number of cells.

Conventional methods for extending cellular lifespan almost invariably alter the phenotypic properties of cells, thereby reducing the value of the immortalized cells. However, the levels of BDNF were decreased at all passages and those of only NGF at late passages (12 and 30), demonstrating that telomerase can stimulate the proliferation and secretion of SCs at early passages, but does not lead to their immortalization. This study also demonstrates that the ability of proliferation and secretion of hTERT-SCs will decrease when they reach a specific point in time.

In summary, we successfully transfected SCs with hTERT and observed that these cells displayed full telomerase activity. Differences in the expression of p53 and p16 prior to and after transfection were not detected. However hTERT alone is not always sufficient to immortalize primary somatic cells. This method can extend the lifespan of the cells without causing cancer-associated changes or alter phenotypic properties.

## Figures and Tables

**Table I.** Primers used for RT-PCR.

Primer	Forward primer	Reverse primer	Ta	Amplicon length (bp)
hTERT	5′-TGTACTTTGTCAAGGTGGATGTG-3′	5′-GTACGGCTGGAGGTCTGTCAAG-3′	68°C	200
p16	5′-CTTCCTGGACACGCTGGT-3′	5′-CGAGGTACCGTGCGACAT-3′	60°C	125
p53	5′-GTTTCCGTCTGGGCTTCT-3′	5′-CCTCAGGCGGCTCATAG-3′	55°C	351
NGF-β	5′-GGCCACTCTGAGGTGCATAG-3′	5′-CATGGGCCTGGAAGTCTAAA-3′	56°C	349
BDNF	5′-AAACCATAAGGACGCGGACT-3′	5′-GATTGGGTAGTTCGGCATTG-3′	56°C	393
β-actin	5′-TCTACGAGGGCTATGCTCTCC-3′	5′-GGATGCCACAGGATTCCATAC-3′	55°C	320
